# Micro-Electromechanical System-Based Parasitic Patch Antenna on Quartz Substrate for High Gain

**DOI:** 10.3390/s25030607

**Published:** 2025-01-21

**Authors:** Haoran Zhao, Qi Wang, Jianyu Du, Lang Chen, Wen Yue, Wei Wang

**Affiliations:** 1 School of Engineering and Technology, China University of Geosciences (Beijing), Beijing 100083, China; 3002200033@email.cugb.edu.cn (H.Z.);; 2 School of Integrated Circuits, Peking University, Beijing 100871, China; wangqi@stu.pku.edu.cn (Q.W.); djy_pku@163.com (J.D.); 2201111447@stu.pku.edu.cn (L.C.); 3 Zhengzhou Institute, China University of Geosciences (Beijing), Zhengzhou 451283, China; 4 National Key Laboratory of Science and Technology on Micro/Nano Fabrication, Beijing 100871, China

**Keywords:** parasitic patch, microstrip antenna, quartz, dielectric loss, MEMS

## Abstract

This paper presents a novel Ku-band parasitic patch antenna based on MEMS technology. The antenna consists of two substrates that are bonded together. The lower substrate houses the main patch and the ground layer, while the upper substrate supports the parasitic patch. To minimize the dielectric loss, the parasitic patch is fabricated using a double-layer suspended film process, combining parylene C and Spin-on-glass (SOG) materials. The two substrates are also bonded using SOG. The proposed antenna achieves a measured bandwidth of 30% (11.1~15.01 GHz), a peak gain of 8.57 dBi, and a compact size of 0.87 × 0.87 × 0.09 λ_0_^3^.

## 1. Introduction

Radio frequency (RF) microsystems leverage advanced micro-nano fabrication technologies to achieve high-density integration of RF, digital, energy, and other subsystems, thereby significantly reducing both volume and power consumption while enhancing overall system performance and reliability [1]. This is inseparable from the large-scale and high-precision processing technology of micro-electromechanical system (MEMS) technology [2]. Silicon (Si) substrates are extensively utilized in the domain of advanced packaging, attributing to their exceptional processing capabilities [3]. However, the relatively high dielectric constant and loss of Si significantly constrain its application in RF microsystems [4].

As a rapidly emerging technology in recent years, glass substrates have garnered significant attention within the field of radio frequency (RF) applications [5]. Glass can be classified into various types based on its composition and manufacturing processes. Fused silica, or quartz, contains almost pure SiO_2_ and no other impurities, ensuring the lowest dielectric constant (Dk) and loss tangent (Df) among glass system materials [6]. Quartz exhibits a high hardness, which consequently impairs its machinability. The Borofloat33 glass, in comparison to quartz, exhibits lower hardness and enhanced processability, making it more suitable for laser-induced deep etching and other fabrication techniques aimed at creating high aspect ratio through-holes. However, its dielectric properties are somewhat inferior in comparison [7]. The photosensitive glass, which can be directly processed via photolithography, shows inferior dielectric properties [8].

Microstrip antennas, the most commonly used antenna in RF microsystems, also offer several advantages, including a low profile, low cost, and ease of integration with planar circuits [9]. However, the traditional microstrip antenna design is often limited by its narrow bandwidth and low gain [10]. Many bandwidth-expanding and gain enhancement technologies have emerged and have been verified. A miniaturized patch-via-wall structure is applied to achieve a large impedance bandwidth of 43.1% [11]. By employing parasitic mushroom type structure, the microstrip antenna owns the advantages of a wide impedance bandwidth of 40% and a high gain of 10 dBi [12].

The parasitic patch improves the directivity and gain of the antenna by loading some parasitic elements around the main patch [13]. The parasitic patch is positioned on the same layer as the main patch, simplifying the manufacturing process; however, the performance enhancement of the antenna remains limited. When the main patch and parasitic patch are placed on separate layers, a significant increase in the antenna gain can be achieved [14].

Glass substrate antennas have been proposed. A glass multilayer stacked 3D scheme enables a filtering antenna with good microwave characteristics and high-density interconnects [15]. A microstrip antenna with glass substrate and parylene C suspended film achieves a high gain of 8.43 dBi [16]. There are no reports of parasitic patch antennas on glass substrates.

Inspired by these works, a novel parasitic patch antenna design on glass substrates has been proposed. The antenna consists of two bonded substrates: the lower substrate serves as a load for both the main patch and the ground plane, while the upper substrate features a hollow structure. A parylene/SOG film is applied on the top surface of the upper substrate, on which a parasitic patch is placed. The antenna is characterized by simulated and measured results.

The article is organized as follows. Section 2 introduces the design and simulation of the parasitic patch antenna. Section 3 describes the fabrication and measurement results of the antenna. In Section 4, the conclusions are summarized.

## 2. Materials and Method

### 2.1. Parasitic Patch Antenna Design

**Figure 1:**
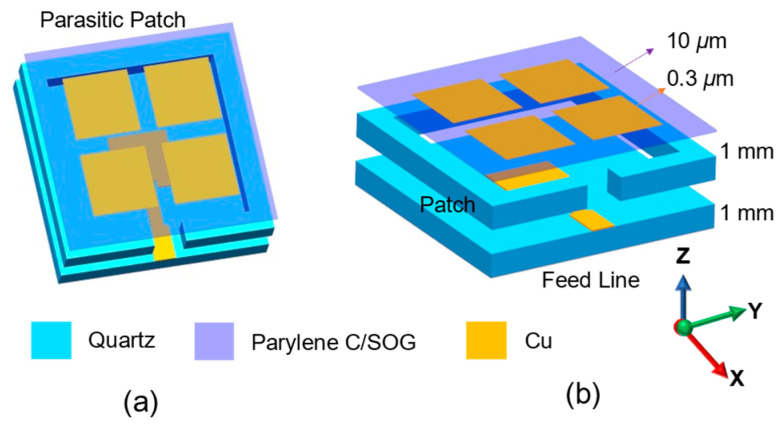
(**a**) Top view and (**b**) side view of the parasitic patch antenna. The coordinate system is shown in the image.

**Figure 2:**
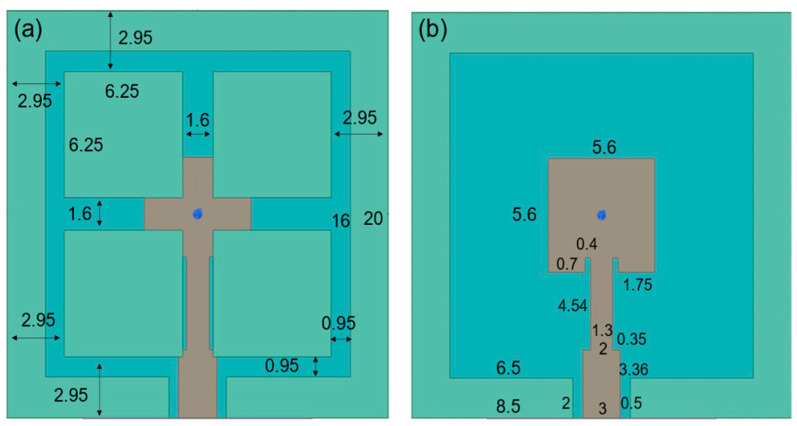
(**a**) Detail dimensions of the parasitic patch; (**b**) main patch. All dimensions are in mm.

Figure 1 shows the structure of the parasitic patch antenna. The upper layer of the complete quartz substrate serves as the main patch, while the lower layer functions as the ground. The main patch is used for feeding the antenna and for radiation excitation. The upper quartz substrate employs a hollow structure to support the parasitic patch. An opening is provided in the side section to facilitate the feed line. Simultaneously, the medium between the parasitic and main patches is air, thereby minimizing the dielectric loss. The parasitic patch consists of a thin 13 μm film of parylene C and SOG. Figure 2 displays the detailed dimensions of the antenna. The materials’ RF characteristics of each part are presented in Table 1. The proposed antenna was designed and optimized with the simulation software ANSYS HFSS 2021 R1.

### 2.2. Comparative Analysis of Parasitic Patch Antenna and Quartz Antenna

**Figure 3:**
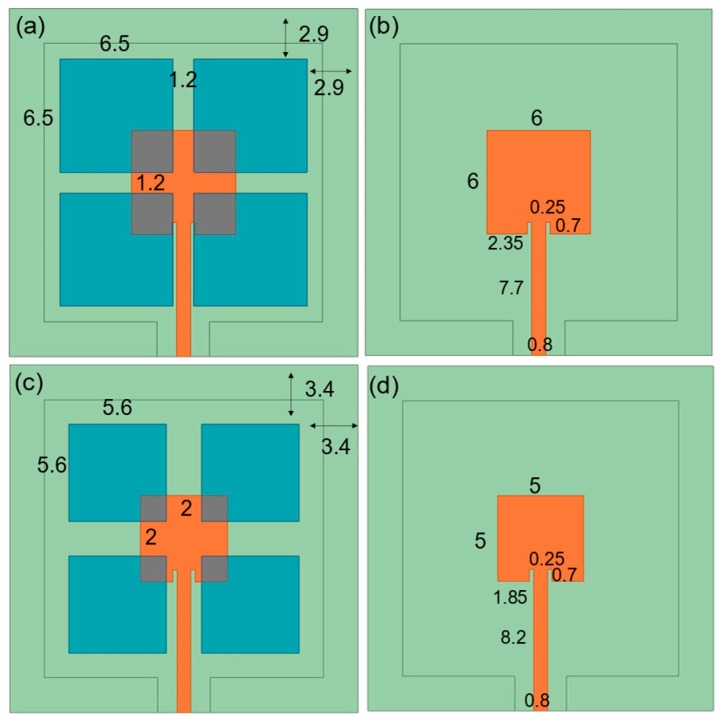
The thickness of the lower substrate is 300 μm, which is the size of the (**a**) parasitic patch and the (**b**) main patch. The 1500 μm lower substrate, the (**c**) parasitic patch and the (**d**) main patch are also shown.

This Section will explore the impact of variations in the dimensions of different components of the antenna on its overall performance. Figure 3 indicates the parasitic patch design with a lower substrate thickness of 300 μm and 1500 μm. For the purpose of comparison, the center frequency is maintained at 13 GHz, consistent with the initial design, even as the thickness is varied. The size and impedance matching design of the main patch must be adjusted as the thickness changes. To optimize coupling at the same frequency and achieve maximum efficiency, the size of the parasitic patch must be slightly larger than that of the main patch. This adjustment ensures effective coupling between the patches, thereby enhancing performance. Better impedance and radiation characteristics can be achieved through the coordinated optimization design of the parasitic and main patches.

**Figure 4:**
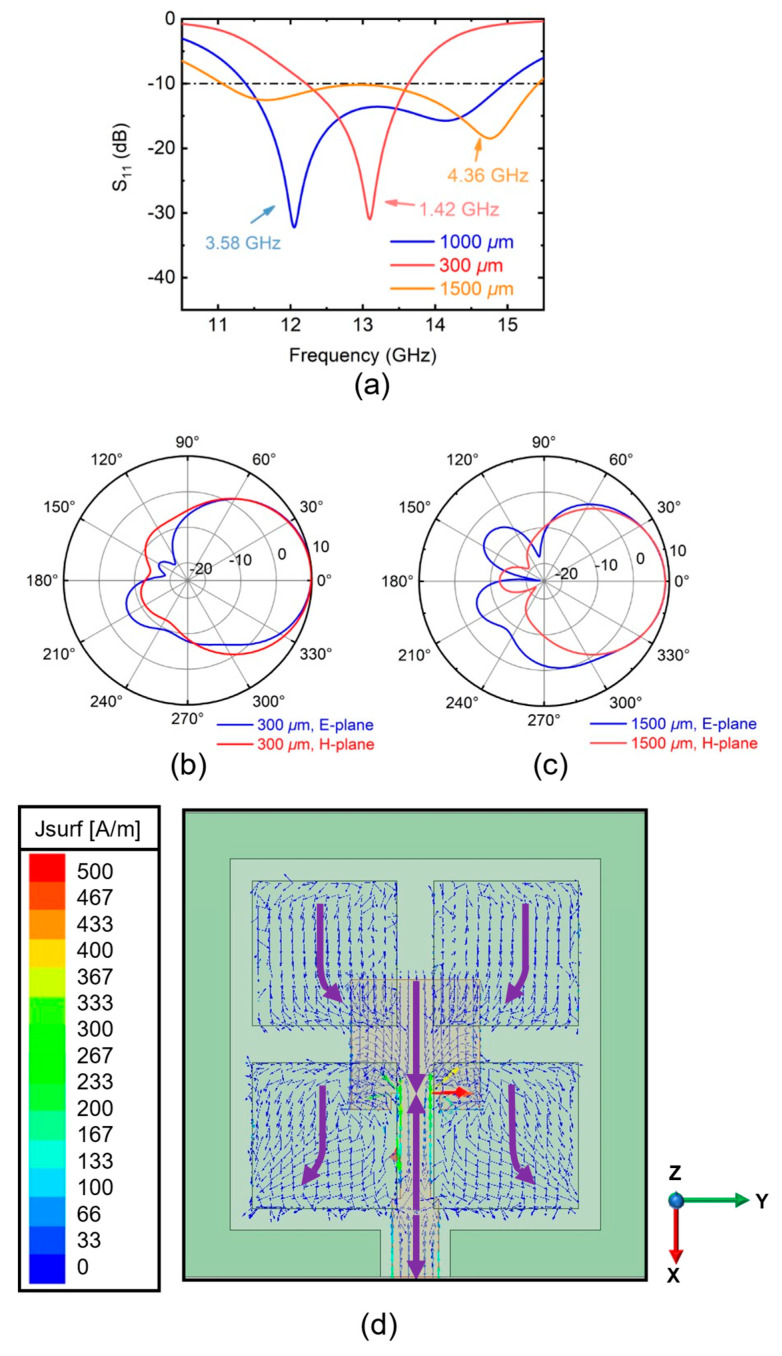
(**a**) Simulated S11 of different thicknesses of the lower substrate. Simulated radiation pattern of the (**b**) 300 μm thickness and the (**c**) 1500 μm thickness substrate at 13 GHz. XOZ plane is E-plane, YOZ plane is H-plane. (**d**) Surface current distribution of parasitic patch antenna.

Figure 4 demonstrates the simulation values of antennas with different lower substrate thicknesses. Bandwidth increases as the substrate becomes thicker. This is consistent with the rule of microstrip antennas. For comparison, only the 13 GHz radiation pattern is taken. The radiation pattern of a 1000 μm thick substrate is discussed in later part. The results of the radiation pattern and peak gain exhibit a similar trend, with the 300-μm-thick substrate showing a marginally higher performance. However, due to experimental conditions and other influencing factors, only samples with a thickness of 1000 μm were fabricated for subsequent measurement and analysis. Increasing the thickness within a specified range can substantially enhance the bandwidth while maintaining a high gain. Practical applications can be optimized by selecting a quartz substrate with an appropriate thickness based on the specific requirements. To explore the bandwidth broadening mechanism of the parasitic patch, the simulation of the surface current distribution is characterized, as shown in Figure 4d. The purple arrow is for reference. Four parasitic patches are symmetrically arranged above the microstrip patch antenna, with a certain distance between them and the microstrip antenna along the Z-axis. A capacitor structure forms between the parasitic patches and the microstrip antenna. Under the excitation of the high-frequency electromagnetic field, this capacitor structure generates a capacitive effect. As a result of this capacitive coupling, a current distribution with the same phase as that on the microstrip antenna is induced on the parasitic patches. From the current distribution, it is evident that the microstrip antenna operates in the TM_01_ mode, which is its primary mode. Similarly, the current distribution on the parasitic patches also follows the TM_01_ mode.

The parasitic patches serve two functions: first, they expand the antenna aperture, which enhances the gain; second, the spatial radiation field created by the in-phase currents on both the microstrip antenna and the parasitic patches leads to energy superposition, further improving the gain. Since the parasitic patches are slightly larger than the microstrip antenna, two adjacent resonances are generated under the same operational mode. The interaction between these two resonances results in bandwidth expansion.

**Figure 5:**
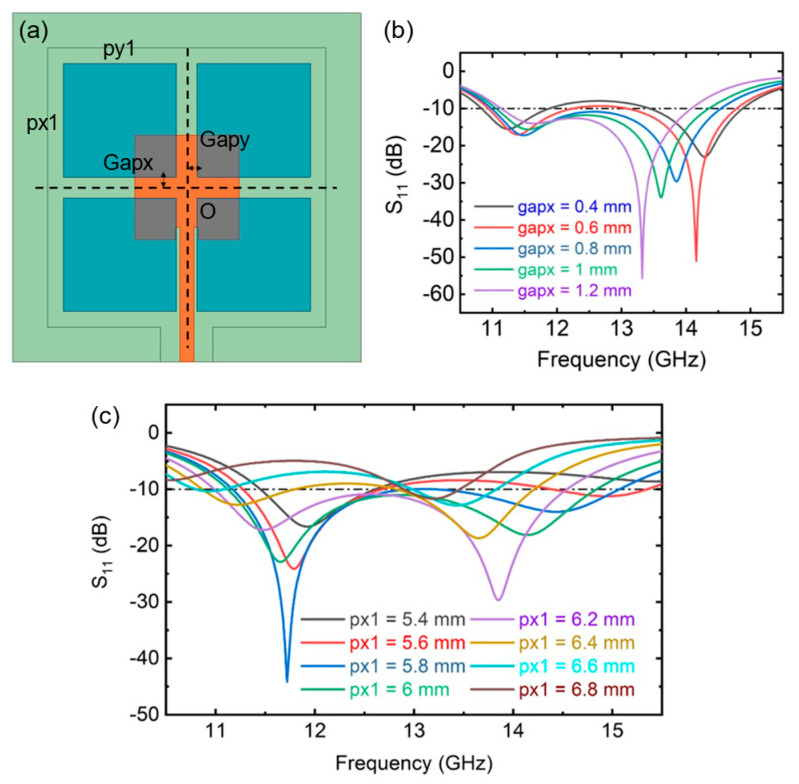
(**a**) Scanning parameter image. (**b**) Simulated S11 of different gapx and (**c**) px1. Only one parameter is varied; the rest of the dimensions remain the same.

Figure 5 indicates the influence of the parasitic patch pattern on the antenna impedance characteristics is further explained. This paper selects only gapx and px1 as the analysis objects. When one parameter is changed, the other dimensions remain unchanged. The spacing and size of parasitic patches can affect the impedance characteristics of the antenna. But the change in parasitic patch size is greater than the effect of spacing.

**Figure 6:**
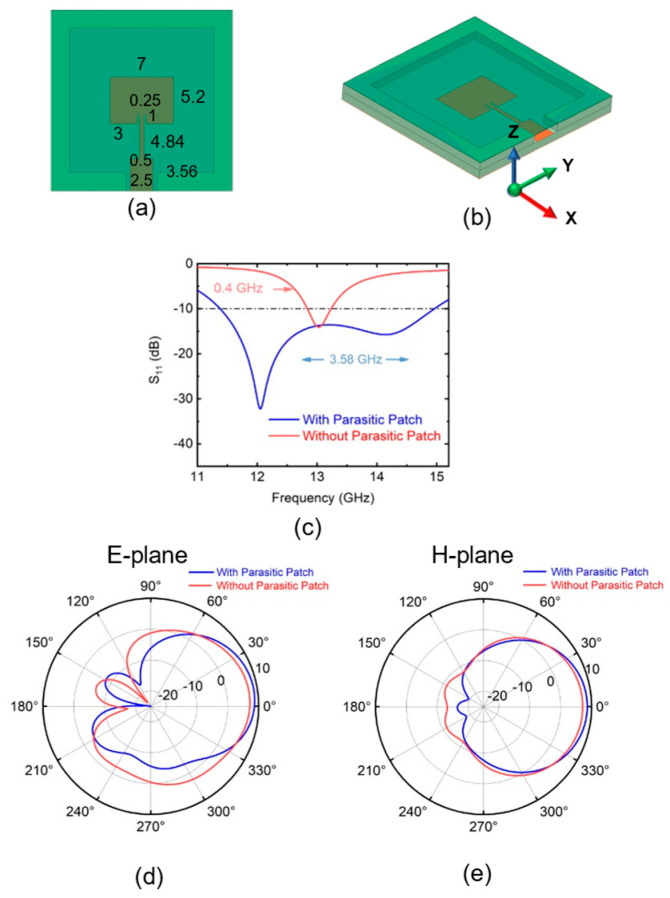
(**a**) Top view and (**b**) side view of the quartz antenna without parasitic patch. (**c**) Simulated S11 of the with and without parasitic patch antenna. Comparison of antennas with and without parasitic patches at 13 GHz on (**d**) E-plane and (**e**) H-plane.

Figure 6a,b exposes the antenna without the parasitic patch. Without incorporating the parasitic patch, it is necessary to redesign the main patch pattern in order to maintain the center frequency at 13 GHz. The bandwidth of the antenna loaded with parasitic patches shows significant improvement compared to the configuration without parasitic patches, as shown in Figure 6c. The peak gain of the parasitic patch antenna at 13 GHz is 8.59 dBi, accompanied by a 3 dB beamwidth of 64°. In contrast, the antenna without the parasitic patch exhibits a peak gain of 7.11 dBi and a 3 dB beamwidth of 76°. The parasitic patch enhances the gain and narrows the beamwidth, resulting in improved antenna directivity.

### 2.3. Fabrication of Parasitic Patch Antenna

**Figure 7:**
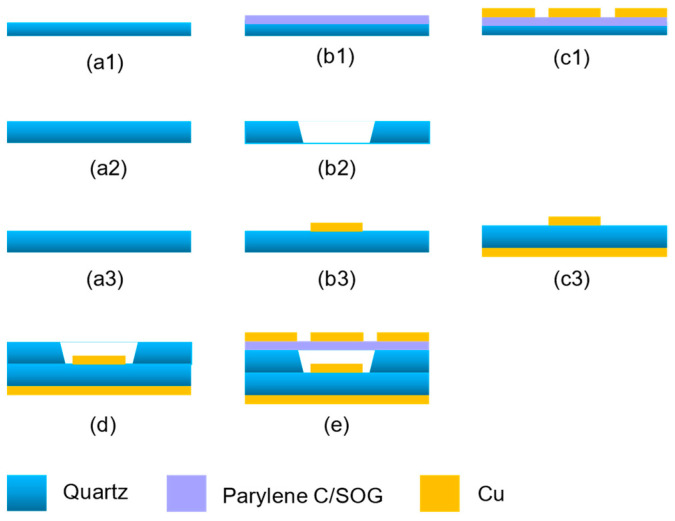
Process flow of the parasitic patch antenna. (**a1**) Quartz wafer 1; (**b1**) Deposit Paralene C film; (**c1**) Deposit Ti/Cu and Patterned; (**a2**) Quartz wafer 2; (**b2**) Laser drilling; (**a3**) Quartz wafer 3; (**b3**) Sputter Ti/Cu and Patterned; (**c3**) Sputter Ti/Cu and Patterned; (**d**) Bonding wafer 2 to wafer 3 by SOG; (**e**) Bonding wafer 1 to wafer 2 by SOG and peeling off wafer 1.

Figure 7 indicates the process flow of the parasitic patch antenna. Three quartz wafers prepare the antenna preparation process. The parasitic patch is prepared by wafer 1. A 10 μm thick of parylene C is deposited on the quartz wafer 1. Then, spin coat 6 μm 4620 photoresist and expose the pattern of the parasitic patch. Subsequently, 200/3000 nm Ti/Cu metal is prepared using physical vapor deposition (PVD). Finally, the graphic metal is prepared by stripping, that is, soaking and removing the photoresist in an acetone solution. The 1 mm thick quartz wafer 2 is used to support the parasitic patch and the main patch. The air cavity is prepared by laser ablation. The main patch is prepared on the quartz wafer 3. The main patch pattern is fabricated following the same procedure as previously described, while the metal layer on the front surface of the wafer is deposited using PVD technique. An additional photolithography step is required at the feeder position to provide protection. Finally, the wafer is subjected to cutting and acetone dissolution to achieve conductivity. After the three wafers have undergone their respective processing steps, they are subsequently bonded utilizing SOG technology. The SOG solution is applied to the wafer surface via spin coating, followed by the bonding of an additional wafer. The assembly is then placed on a hot plate and subjected to a thermal treatment at 130 °C for a duration of 1 h. After the bonding process is completed, a release agent is applied to facilitate the detachment of perylene C and wafer 1. The mechanical properties of parylene C and SOG bilayers, along with the characterization of the bonding properties between SOG and quartz substrates, have been extensively explored in the existing literature [16].

**Figure 8:**
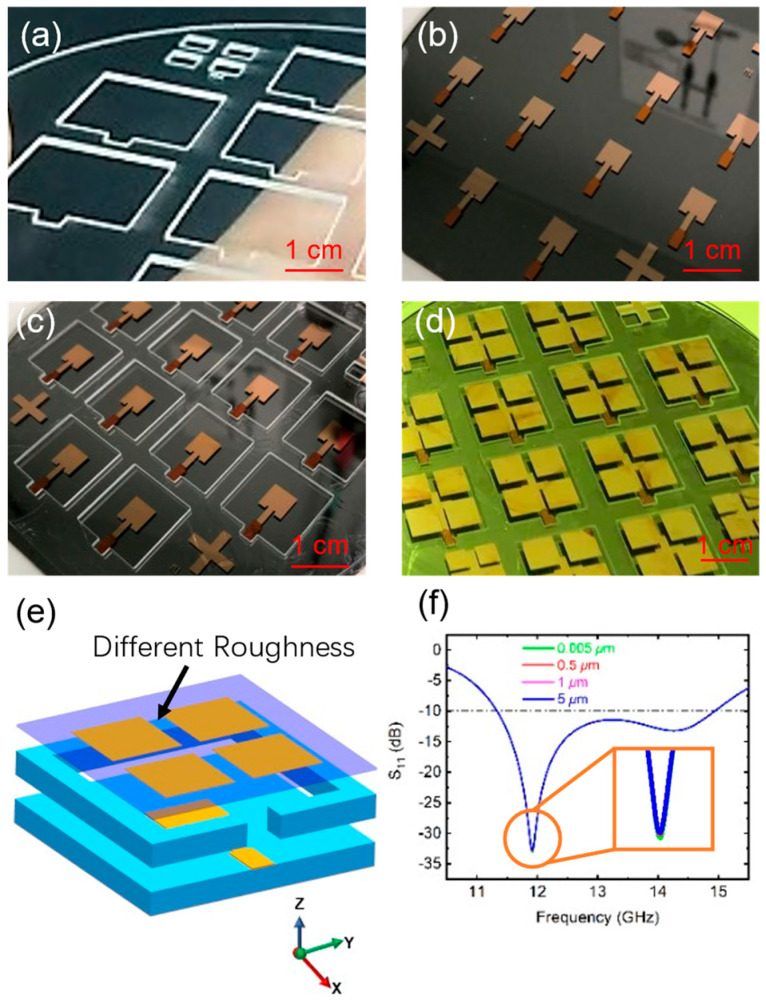
(**a**) Wafer 2 surface modified by laser ablation. (**b**) The prepared wafer 3 exhibits the main patch pattern on its surface, with the backside coated in photoresist. The photoresist layer at the feed line of the main patch provides a protective function. (**c**) Wafer 2 is bonded to wafer 3 by SOG. (**d**) Fabricated parasitic patch antenna wafer. (**e**) Different roughness of parasitic patch films. (**f**) Simulated S11 of the parasitic patch at different surface roughness.

Figure 8 presents the photos during the processing of the parasitic patch antenna. Figure 8d shows the prepared antenna wafer. By cutting it, a complete antenna unit can be obtained. Since the parasitic patch antenna is fabricated on a suspended film, the roughness of the film and other fabrication errors must also be considered. Figure 8e,f illustrates the analysis of how the roughness of the parasitic patch antenna impacts the S11 parameter. The results demonstrate that within the roughness range of 0.005 μm to 5 μm, the performance of the antenna remains largely unaffected. The presence of obvious delamination or film rupture should be regarded as antenna failure rather than a mere error. As for factors such as vibration and high acceleration, which may occur in practical applications, the article did not include relevant experimental analysis. These aspects will be addressed in future research.

## 3. Results and Discussion

**Figure 9:**
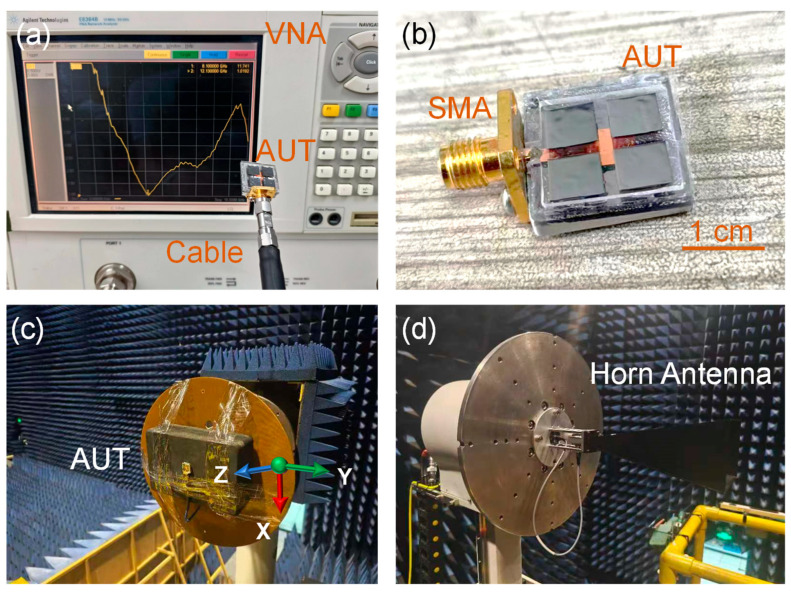
(**a**) The AUT and VNA. (**b**) The parasitic patch antenna with SMA. (**c**) The AUT in a dark room. (**d**) Standard horn antenna for measurement.

Figure 9a exhibits the vector network analyzer (VNA) and the antenna sample. The VNA is used to measure the S11 and voltage standing wave ratio (VSWR) of the antenna. The subminiature version a (SMA) connector is connected to the feed line and wrapped with conductive glue to increase conductivity, as shown in Figure 9b. The flatness of the antenna sample surface after cutting is somewhat inadequate, and the film in the region of the parasitic patch shows visible wrinkles. This issue is caused by the excessive thickness of the substrate, which leads to damage during the mechanical dicing process. Future research should focus on further optimization of the dicing process to minimize these defects. Figure 9c exposes the antenna under test (AUT) in a dark room. The gain and radiation pattern of the antenna are tested in a dark room. A standard horn antenna is used as a reference signal source to help measure the radiation characteristics of the AUT, as illustrated in Figure 9d.

**Figure 10:**
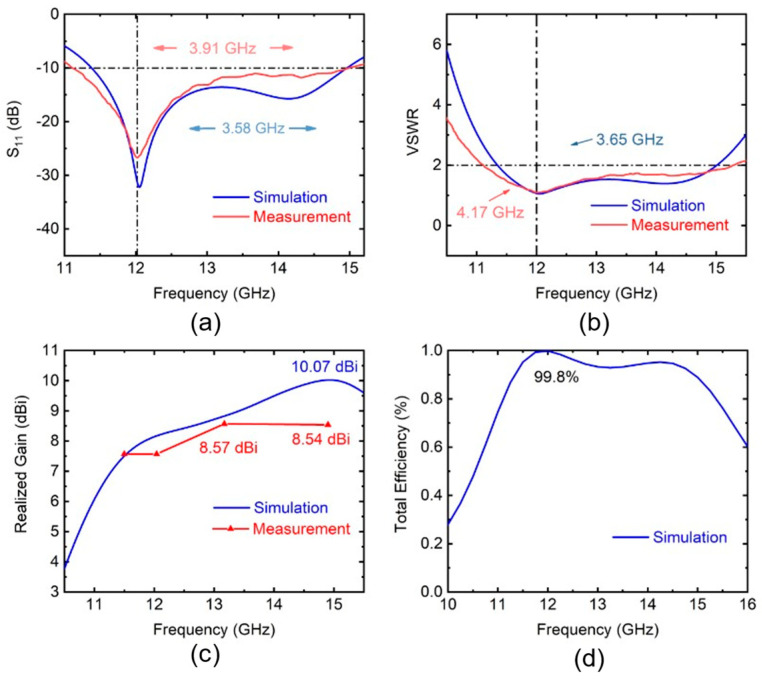
(**a**) Simulated and measured S11 and (**b**) VSWR of the parasitic patch antenna. (**c**) Simulated and measured values of peak gain at different frequencies. (**d**) Simulated total efficiency of the antenna.

Figure 10a demonstrates the simulated and measured S11 of the antenna. The minimum values of both are at 12 GHz. The simulated bandwidth was estimated to be 3.58 GHz, whereas the measured bandwidth was observed to be 3.91 GHz. Figure 10b shows the VSWR value by simulation and measurement. The frequency range over which the simulated VSWR remains below 2 is 3.65 GHz, whereas the corresponding measured range is 4.17 GHz. The bandwidth corresponding to a VSWR of less than 2 is similar to the bandwidth where S11 is below −10 dB. Both the simulated and measured results exhibit this characteristic, indicating that the test outcomes align with the expected values.

Figure 10c indicates the relationship between peak gain and frequency. The simulation results demonstrate that the peak gain exceeds 6 dBi within the frequency range corresponding to the bandwidth, with the maximum reaching 10.07 dBi. The measured peak gain reaches a maximum of 8.57 dBi at a frequency of 13.17 GHz. The measured and simulated values of S11 and VSWR exhibit a high degree of consistency, whereas the gain shows a noticeable discrepancy. This can be attributed to the fact that the impedance characteristics of the antenna are predominantly governed by the main patch and the underlying quartz substrate. The quartz substrate is highly stable and exhibits excellent surface flatness. The radiation characteristics of the antenna are primarily influenced by both the parasitic patch and the main patch. The parasitic patch, being a thin-film structure, is susceptible to deformation due to process variations during fabrication. This deformation may lead to discrepancies between the simulated and measured peak gain. Figure 10d presents the simulated total efficiency of the antenna, and the maximum is about 99.8%. We believe that the parasitic patch can also improve the antenna’s efficiency, but due to experimental conditions, this efficiency has not been experimentally verified and will be supplemented in subsequent work.

**Figure 11:**
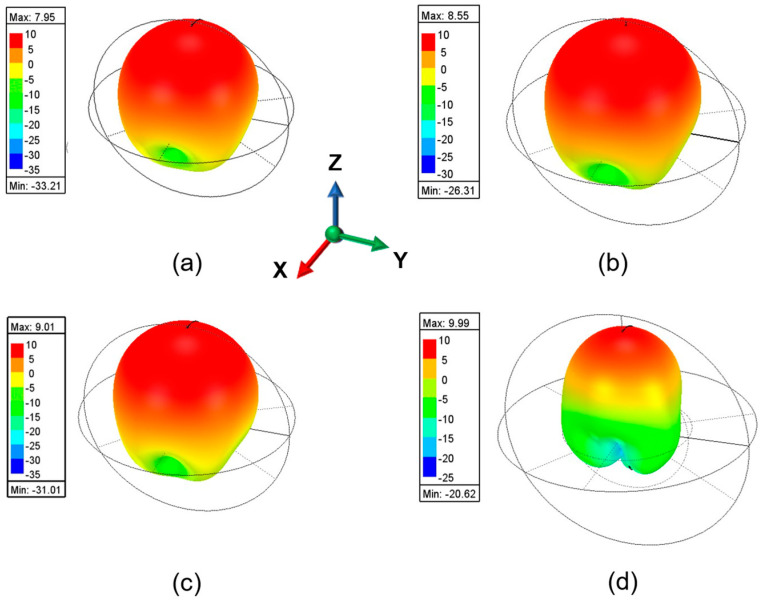
(**a**) Simulated 3D radiation pattern at (**a**) 11.5 GHz; (**b**) 12.04 GHz; (**c**) 13.17 GHz and (**d**) 14.9 GHz.

Figure 11 indicates the simulated 3D radiation pattern at different frequencies. The peak gain is observed in the direction of the Z-axis. The intensity of back radiation is relatively low.

**Figure 12:**
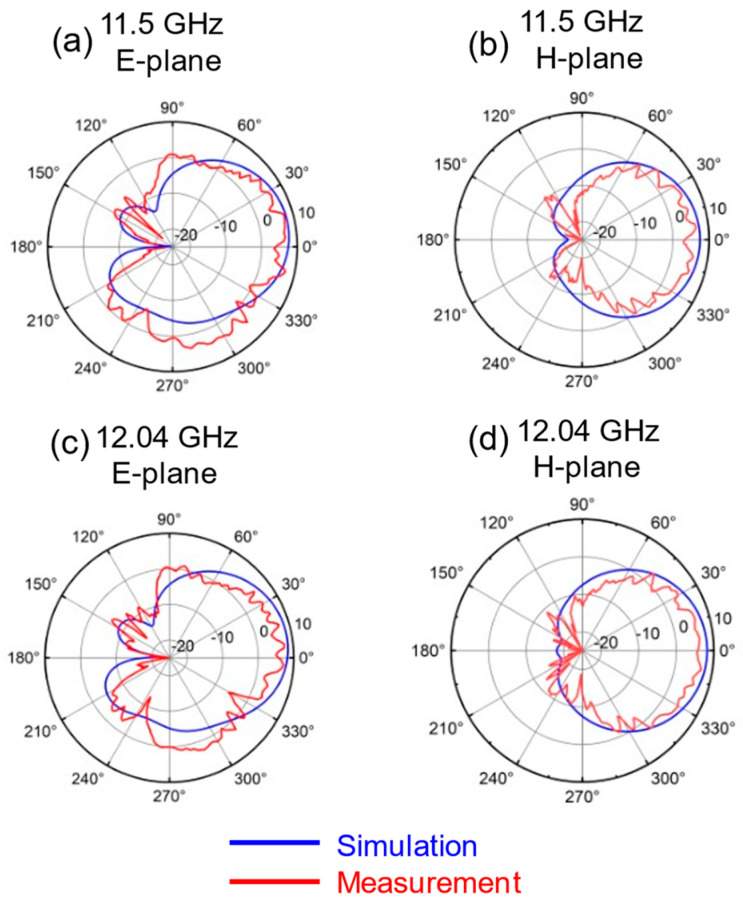
Simulated and measured radiation pattern at (**a**) 11.5 GHz on E-plane; (**b**) 11.5 GHz on H-plane; (**c**) 12.04 GHz on E-plane; (**d**) 12.04 GHz on H-plane. The XOZ plane is E-plane, the YOZ plane is H-plane.

**Figure 13:**
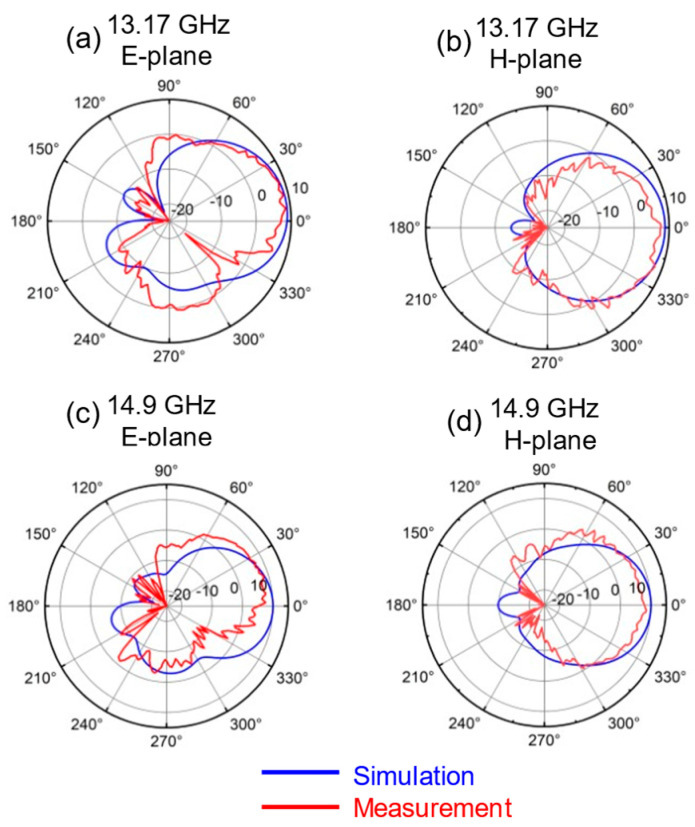
Simulated and measured radiation pattern at (**a**) 13.17 GHz on E-plane; (**b**) 13.17 GHz on H-plane; (**c**) 14.9 GHz on E-plane; (**d**) 14.9 GHz on H-plane.

Figure 12 and Figure 13 show the simulated and measured radiation pattern at different frequencies. The simulation and measured values of the H-plane are in good agreement. There is a certain inconsistency between the two values in the E-plane. The error in the E-plane is increased due to the installation of the connector and the influence of the fixture. The measured values of the radiation pattern exhibit spikes, which may be attributed to insufficient accuracy of the testing system or inadequate sampling resolution.

**Figure 14:**
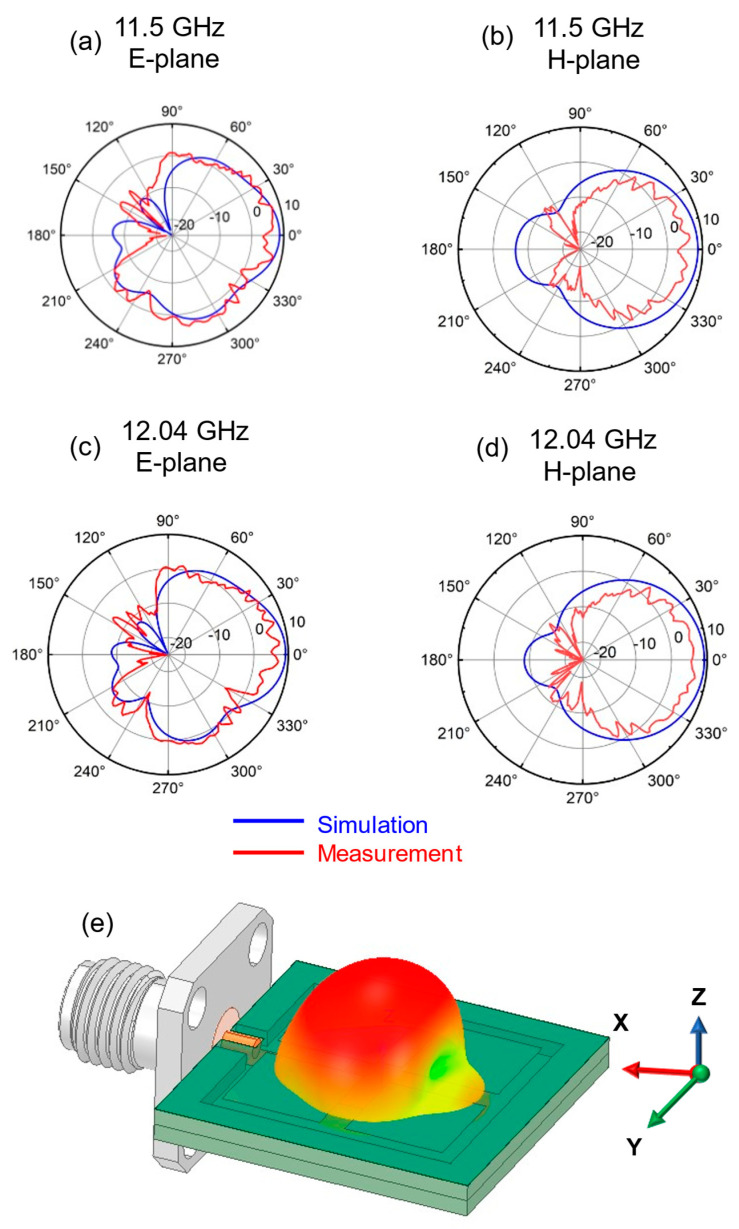
Simulated and measured radiation pattern by loading SMA connector at (**a**) 11.5 GHz on E-plane; (**b**) 11.5 GHz on H-plane; (**c**) 12.04 GHz on E-plane; (**d**) 12.04 GHz on H-plane. (**e**) Parasitic patch antenna model loading SMA connector.

**Figure 15:**
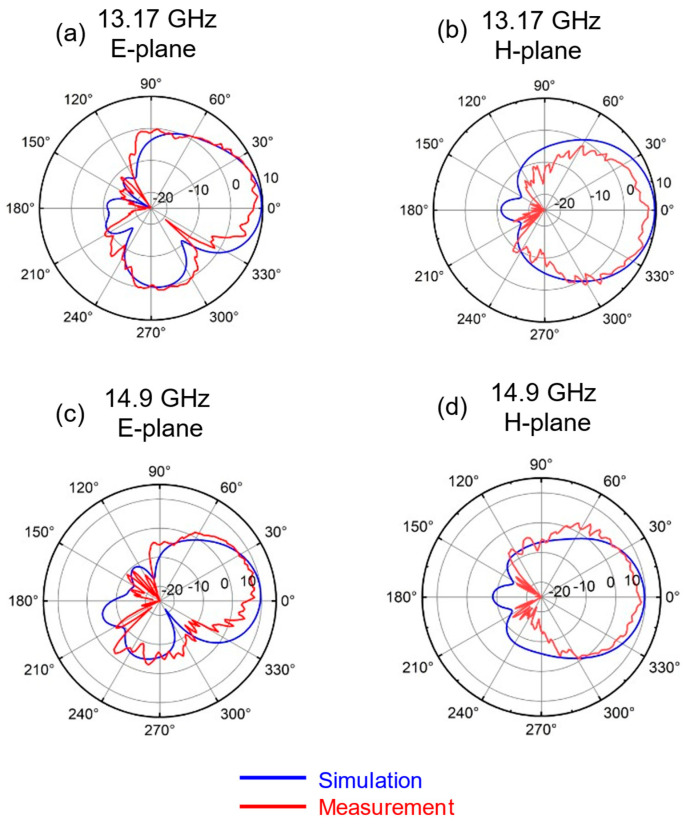
Simulated and measured radiation pattern by loading SMA connector at (**a**) 13.17 GHz on E-plane; (**b**) 13.17 GHz on H-plane; (**c**) 14.9 GHz on E-plane; (**d**) 14.9 GHz on H-plane.

There is a discrepancy between the measured radiation pattern and the simulation results. To address this, the author incorporated the measured SMA connector of the same model and size into the ANSYS HFSS simulation model. This adjustment resulted in a radiation pattern that closely matches the measured values, as shown in Figure 14 and Figure 15. The model of the SMA connector is depicted in Figure 14e. It also shows a 3D radiation pattern simulation at 13.17 GHz. Due to the small size of the antenna and the large size of the connector flange, electromagnetic waves will be reflected, disrupting the current distribution on the ground. This results in an uneven current distribution, which in turn causes distortions in the radiation pattern.

Table 2 presents a comparison of recently reported antennas and glass antennas. The reported antenna [17,18] was constructed with a glass substrate, albeit with a lower gain. The reported work [19] was also made with a glass substrate and shows high gain, but the author considers that the processing of the device poses considerable challenges. The antennas reported in refs. [20,21] were fabricated using print circuit board technology, resulting in a compact design. However, they demonstrated relatively low peak gain.

## 4. Conclusions

In this article, a novel Ku-band parasitic patch antenna on a quartz substrate using MEMS technology was designed and fabricated. The antenna consists of two bonded substrates, with a suspended film process employed between the parasitic patch and the main patch. This approach effectively reduces substrate loss and enhances the radiation efficiency. The advantages of the proposed parasitic patch design are verified through detailed simulation analysis. The antenna achieves a measured bandwidth of 30% and a high gain of 8.57 dBi. A comparison with the reported work shows that the antenna achieves high gain and low profile. Although discrepancies exist between the measured results and the simulated values, these can be addressed through process optimization and by evaluating the accuracy of the test system. This study also simulated the antenna with a SMA connector, and the results demonstrated good consistency with the measured values. These findings offer valuable insights into the relationship between the side-fed microstrip antenna and the connector.

## Figures and Tables

**Table 1:** Materials’ RF characteristics.

Materials	Dielectric Constant (D_k_)	Loss Tangent (D_f_)
Parylene C	2.95	0.02
SOG	3.0	0.001
Quartz	3.75	0.0002
Air	1	0

**Table 2:** Comparison of recently reported glass-based antennas and other antennas.

Operating Band (GHz)	Area/ Thickness (λ_0_^2^/λ_0_)	Peak Gain (dBi)	Materials and Process
26~30	4.67 × 4.67/0.3	5	Glass/Machining [17]
58~62	~0.6 × 0.6 × 0.06	9.1	Glass-based Air Cavity [19]
55~65	~1.38 × 1.38/0.33	8.5	Glass-based Air Cavity [18]
10	0.67 × 0.53/0.027	6.3	RO-4003(C) [20]
9.7 11.4	0.32 × 0.32/0.05	4.4 3.2	FR4 [21]
11.1~15.01	0.87 × 0.87/0.09	8.57	Quartz-based (Proposed)

## Data Availability

Data are contained within the article.
